# Mental Healthcare Consumers’ Experiences of Mental Health Care: Variation in Knowledge by the Family Members and Support

**DOI:** 10.1177/23779608241272489

**Published:** 2024-08-09

**Authors:** Mihloti E. Shimange, Hilda Nwamuhohova Shilubane, Nthomeni Dorah Ndou, Adrinah Seani Mulondo

**Affiliations:** 1 Department of Advanced Nursing Science, 56868 University of Venda, Thohoyandou, Limpopo, South Africa

**Keywords:** consumers’ experiences, knowledge disparity, family members

## Abstract

**Introduction:**

Knowledge and support by members of the family towards the care of the consumers of mental health services is the core priority and is noticeable by the users as it gives and eases the life of the users during therapy and rehabilitation. However, there have been documented instances of families that neglect their relatives with mental illnesses.

**Objective:**

This study explored the experiences of mental healthcare consumers regarding family members’ knowledge of mental disorders and support.

**Methods:**

Participants who were granted leave of absence were selected through nonprobability, purposive sampling. Data were collected using face-to-face unstructured discussions. Data were analyzed using Colaizzi's technique.

**Results:**

Findings revealed misconceptions versus insight on the cause of mental disorders, knowledge deficit on the effect of treatment, poor support from family members, financial challenges perceived as a source of poor support, and lack of psychological support and its consequences. Mental healthcare consumers verbalized limited support from family members. They reported variation in terms of family members' knowledge of their mental condition.

**Conclusion:**

Training family members on mental health illness is critical to the future of health care as there will be no misunderstanding between them and the consumers of mental health care. Healthcare consumers’ feelings of sadness and anxiety could be avoided by avoiding conflicts over their social grants. The government should invest in assisting family members of mental healthcare consumers.

## Introduction

Individuals with a diagnosis of mental illness are estimated to be 970 million by the World Health Organization [WHO] (2022). Globally, mental diseases have become increasingly common and were the seventh leading cause of disability-adjusted life years in 2019. Mental disorders were the second leading cause of years lived with disability worldwide in both 1990 and 2019 (GBD 2019 Mental Disorders Collaborators, 2022) and a wide variation in receiving evidence-based care (Wood et al., 2019). Persons suffering from mental disorders need support from society, such as support in establishing and maintaining personal, familial, and social connections.

Members of the family have a critical part in consumers' long-term treatment strategies. When family members are well informed about the condition, its symptoms, and management strategies, and involved in healthcare services, they can provide better support to patients (Babaei & Abolhasani, 2020). Families' care and support are essential to the healthcare recipient's well-being, particularly for individuals with mental illnesses. Family members are fundamental associates in mental health care, according to WHO (2022). They are essential in supporting mental health patients emotionally, assisting them in getting suitable medication, and speaking up for their needs in the medical system. Takhar and Hyden (2020) stated that mental health changes in wealthy countries have led to a move from hospital to home care. Family members play a vital part in patient care by contributing, assisting with home care, and addressing patient expectations.

Support of family members is an essential component of care centered around patients, influencing the provision of quality care and outcomes for patients (Jazieh et al., 2018); and the consumers of mental health services require substantial support from family members, notably spouses, siblings, and children (Raluthaga et al., 2023a).

The family should be aware that odd behaviors exhibited by a person suffering from a mental illness are symptoms of the illness rather than deliberate acts (Chronister et al., 2021). It is critical to teach the relatives of those suffering from mental illnesses to prevent annoyances and conflicts. The difficulties and tensions associated with mental health treatment are lessened when families are informed and involved in the process. Allowing mental healthcare consumers to express their opinions on their care provides them peace and insight because there is a podium to communicate their troubles. Consumers highlight the necessity of psychiatrists communicating mental diagnoses to mental healthcare consumers and their family members while also giving them courage and knowledge regarding their condition in helping family caregivers become more confident and competent providers (Ong et al., 2021).

## Review of Literature

Research revealed that to achieve quality outcomes, anyone in the healthcare sector must participate. A partnership involving consumers of mental health care, mental health practitioners, health professionals, family members, and scholars is required to enhance practices and health outcomes for users requiring care (Singh et al., 2019). Patients should actively participate in the design of interventions, adopting an empowered role in their care. Lay healthcare supporters should increase their professional knowledge to properly enable family based interventions. Medical practitioners should not only provide professional support but also emotional support and convenient services to patients and their families (Fu et al., 2024).

The participation of family members in caring for severely sick adult inpatients is common (Akpan-Idiok et al., 2020). Family members have essential roles in patient care, including aiding the healthcare team with care delivery, enhancing the safety of patients and the standard of care, and participating in care at home (Jazieh et al., 2018). It is commonly agreed that to improve services and care, patients' personal experiences, as well as those of their family and friends, must be acknowledged and sought to be included in care planning and organization (Svendsen et al., 2021).

The significance of relatives in the care of patients with mental disorders is generally recognized, and what is required is an agreement from service users' views (Maybery et al., 2021). Previous research has been carried out to determine the knowledge of caregivers of mentally ill patients (Birkie & Anbesaw, 2021; Gabra et al., 2020; Nabi & Rizvi, 2022; Sandi et al., 2020) and their support (Aass et al., 2022; Raluthaga et al., 2023a). Furthermore, studies on family members (Chronister et al., 2021; Raluthaga et al., 2023b) and healthcare practitioners' perspectives (Mpheng et al., 2022) related to caring for mental healthcare users (MHCUs) have been conducted and little is known about the users' experiences of family members’ knowledge and support. Hence, this study's main objective was to explore the mental healthcare consumers' experiences of family members' knowledge and support.

## Methods

### Setting and Research Design

The experiences of mental healthcare consumers regarding family members’ knowledge of mental illness and support were investigated using a qualitative phenomenological approach. This design permitted the researchers to explore and describe mental healthcare consumers' life encounters (Alhazmi & Kaufmann, 2022; Polit & Beck, 2021).

The mental health institutions in Limpopo province were the study setting. The researcher chose these institutions as they are the only specialized mental hospitals in the province. This province is located in South Africa's northernmost region. It has borders with Botswana to the west, Zimbabwe to the north, and Mozambique to the northeast. Within South Africa, Limpopo is bordered by the provinces of Mpumalanga to the southwest, Gauteng to the west, and the Northwest to the south. The provincial capital is Polokwane, formerly known as Pietersburg. Limpopo is celebrated for its diverse landscapes, encompassing savannahs, mountains, and bushveld. This province boasts a wealth of wildlife and is steeped in cultural heritage (Britannica, 2024).

### Research Question

The following research question was addressed in this study: What are the experiences of mental healthcare consumers regarding the knowledge of mental illness and support by family members?

### Sample and Sampling

The study population comprised 13 MHCUs of whom 10 were males and three females. To choose participants, purposive sampling was used. Fifteen participants were approached face-to-face by the researcher to take part in the study. On behalf of the researcher, the operational managers of the wards recruited the participants from the selected mental health establishments. Mental healthcare consumers who were stable and able to communicate were recognized by operational managers.

### Inclusion/Exclusion Criteria

Participants had to be at least 18 years old, have a mental disorder diagnosis, recently had a leave of absence (LOA) from the institution, and be admitted to a mental health facility to be included in the study. Recent LOA was included to ensure MHCUs experienced the knowledge of their family members about mental illness as well as their support while at home. Participants younger than 18 years were excluded because they were not legally competent to give informed consent.

### Data Collection

After the mental healthcare consumers had been identified, participants were personally approached and asked to take part in the research project. The researcher coordinated and conducted the interviews with potential subjects and got approval from the nurse in charge. The discussions with participants took place from October to December 2022 in a private consulting room and were coordinated by the first author, a female doctoral degree candidate, and a psychiatric professional nurse. All the participants were told before the interview of the goal of the study and that they would participate at their free will, with no consequences should they choose to withdraw from the discussion. The confidentiality of their answers was assured. Participants were aware of the interview length beforehand. The first author and the participant spoke for between 35 and 50 min in a one-on-one interview in the private consulting room. There was no participant pull-out, and the researcher did not know the participants beforehand. The discussions were recorded on tape, and field notes were taken at all times. The first author asked the participants one pretested question using the local language, and follow-up questions evolved as the interviews progressed from the fundamental topic: what are your experiences regarding family members’ knowledge of mental illness and support? Since the study used unstructured interviews, there were no specific follow-up questions prepared beforehand. The follow-up questions were based on the responses of the participants. Discussions continued up to the 13th MHCU due to data saturation and no repeat interviews were conducted. The transcriptions were made accessible to the participants, but nobody read them.

### Data Analysis

Thematic analysis using an inductive approach was used. This focuses on individuals' experiences, perspectives, and perceptions, generally aiming for a thorough knowledge of people's lived realities. The goal is to uncover themes that provide insight into a specific group or culture. Original conversations and written notes were directly typed into Microsoft Word. The data were examined through Colaizzi's technique (Praveena & Sasikumar, 2021). The Colaizzi method was chosen because it enabled new knowledge to be revealed and gain insights into the lived experiences of MHCUs regarding their family members' knowledge and support. Once the text has been prepared for individual transcripts, the first author reads each transcript multiple times to acquire a thorough grasp of the mental healthcare consumers' interactions with family members' care. Memos were kept to further immerse the researcher and underline key themes until the researcher was acquainted with what was being investigated. The themes were identified by the first author. Within each theme, subtopics were searched for, and appropriate quotations that conveyed the main idea were chosen. To assess the distinct component of the experiences, statements that did not describe the MHCU experience were reduced or eliminated. This procedure entailed determining whether the statement contained an essential moment to understand the experience and whether it could be labeled. Clustering was used to group related constituents, and each category was assigned a theme title. This process was carried out numerous times to aggregate and minimize groups until all constituents were divided into two themes and five subthemes. The themes and subthemes were confirmed by an independent researcher.

#### Trustworthiness

Trustworthiness was maintained throughout the investigation by employing the following Guba's four criteria summarized by Creswell and Creswell (2022). All criteria were met as follows: Participants remained in the field until data saturation was achieved, which ensured the study's legitimacy. It was further established by audibly summarizing the participants' comments, which they verified. The researchers' verbatim transcriptions from audiotape recordings and field notes, leaving no material out ensured transferability. The study's findings were compared to the current literature as a way of ensuring confirmability. The study's methodology was described, interviews were verbatim transcribed, and data were analyzed. To ensure rigor of procedures and data quality, the purpose of the study was explained to participants before the interviews. The first author spent time with participants to establish rapport before the commencement of individual interviews at the agreed-upon time and venue. Member checks were completed. The interview transcripts were verified for completeness and accuracy by the participants to ensure that they accurately reflect the content and intent of their contributions.

## Ethical Consideration

The University's Ethical Committee gave the study ethical approval and permission from the provincial Department of Health and associated institutions was obtained. All participants gave verbal consent before undertaking the research. The voluntary participation in the study was highlighted and they could leave when they felt like, with no consequences. The participants' rights to autonomy, confidentiality, privacy, and fairness were upheld.

## Results

More participants were males (n = 10) than females (n = 03) with an age range of 24 and 66 years. Five were aged 20–35, two were between 40 and 45 years, those between 50 and 59 years old were four, two were 60 years and above, and the grades passed ranged from 2 to tertiary level. For details see Table 1.

**Table 1.** Outline of the Participants’ Characteristics.

Participant	Gender	Age	Education	Diagnosis	Year diagnosed	Caretaker
01	Male	24	Grade 12	SIPD	2017	Parents
02	Male	42	Grade 11	BMD	2014	Mother
03	Female	52	Grade 4	Schizophrenia	2006	Spouse
04	Male	62	Grade 2	Schizophrenia	1977	Siblings
05	Male	66	Grade 11	SIPD	2020	Parents
06	Male	44	Grade 9	SIPD	2010	Mother
07	Female	35	Grade 4	BMD	1998	Siblings
08	Male	26	Grade 10	Schizophrenia	1994	Mother
09	Male	23	Grade 08	SIPD	2016	Parents
10	Female	56	Grade 2	Schizophrenia	1984	Spouse
11	Male	35	Grade 4	Schizophrenia	2006	Siblings
12	Male	59	Grade 1	SIPD	1994	Siblings
13	Male	56	Teacher	SIPD	1998	Kids

Abbreviations: SIPD = substance-induced psychotic disorder; BMD = bipolar mood disorder.

### Themes and Subthemes Emerging From the Data Analysis

The following two themes emerged as experienced by mental healthcare consumers concerning family members' knowledge of mental illness and their support as presented in Table 2. The themes include (1) comprehension of mental disorders and the effect of treatment and (2) MHCUs need support from family members. These are some findings of the ongoing project.

**Table 2.** A Summary of the Themes and Subthemes.

Themes	Subthemes
1. Comprehension of mental disorders and the effect of treatment	1.1 Misconceptions versus insight on the cause of mental illness or disorder
	1.2 Knowledge deficit on the effect of treatment
2. Mental healthcare users need support from family members	2.1 Poor support from family members
	2.2 Financial challenges perceived as a source of poor support
	2.3 Lack of psychological support and its consequences

### Theme 1: Comprehension of Mental Disorders and the Effects of Medication

Knowledge of a mental condition by the patients and their family members improves good relationships and trust between them and health outcomes. On the contrary, poor knowledge of the condition contributes to poor compliance with treatment by mental healthcare users. Two subthemes that emerged under this theme are misconceptions versus insight on the cause of mental illness and knowledge deficit on the effect of treatment.

#### Subtheme 1.1: Misconceptions Versus Insight on the Cause of Mental Illness

Misconceptions are incorrect views based on faulty understanding. Participants appeared to have a rudimentary grasp of the causes of mental disorders. This is what they had to say,

In 2005, the woman who was married to my stepfather before he married my mother, bought two school shirts for me and my brother. My mother told my brother not to wear those shirts. She has been shown in her dream that those shirts have something in them. From nowhere, the woman told my grandmother who is a traditional healer, that I was bewitched and required treatment. (P11, M)

I don’t want to lie, I don’t know what was happening to me, it was not the first time I beat her, even the last time I was admitted because I beat her. I don’t know why the person who bewitched me wants me to kill my mother so that I can suffer because I can see that this thing wants me to fight with my mother. (P1, M, 23)

I had a boil in my hand when I first felt unwell. I had a wound and my hand was swollen. I then became confused, and they rushed me to the nearest hospital. That is how it started. We are surrounded by witches but fortunately, my mother prays a lot hence their mission failed. (P2, M, 26)

In contrast, one participant demonstrated the insight the family members have regarding mental conditions. He mentioned that his family members are aware that his mental condition was caused by taking alcohol and other substances. The following narrative attests to this:

My siblings know about my mental condition except that I now have diabetes mellitus which I was diagnosed recently together with HIV. Sometimes they will say that I caused this to myself because I was taking drugs and alcohol. (P12, M)

#### Subtheme 1.2: Knowledge Deficit on the Effect of Treatment

When a person lacks understanding or feels confused about the condition or treatment of their relatives with mental illness, it can hinder their ability to make informed decisions and actively participate in patient care. Participants outlined their concerns below regarding their treatment,

Health professionals never called my sister to speak to her or explain anything concerning my condition. When she is here to see me, they just greet her and that is all. Then they will call me to stay with her and chat, when we finish our conversation, she just bids them farewell and leaves. When I ask for money to go to a check-up she refuses because she does not understand the importance of taking treatment when you are a psycho because of little knowledge of mental condition. (P13, M)

Ha … he treats me well because sometimes he reminds me about my treatment. The problem arises when his kids tell me that I shouldn't take medication, then I start to get confused because he says I should take treatment when on the other hand his children tell me not to because I am not sick. (P2, M)

Sometimes you find that I take treatment very late or end up not taking it because my uncle prioritizes household duties more than taking treatment. Once we start doing household duties, he does not allow me to have a break, he tells me that I will take treatment when we are done with what we are doing. (P4, M)

I say that they don’t want to accept my mental condition because whenever am at home and it's time for me to go for a check or treatment collection, they will say they don’t have money and start blaming me and even telling me that they are not my parents. (P12, M)

### Theme 2: MHCUs Need Support From Family Members

The main component of a solid relationship and good mental health is often deemed to be social support. Social support is basically about having a network of family and friends who you can use in times of need. Positive health outcomes are associated with strong family relationships. Participants reported receiving poor support in terms of practices, finances, and emotions from their relatives.

#### Subtheme 2.1: Poor Support by Family Members

In this study, participants expressed negative experiences of nonsupportive family members. They stated limited to complete lack of support from their family members whether during admission to the mental health establishment or when at home. Participants indicated their feeling of being forgotten as family members do not support them. One participant further stated the only time he received visitors was when the healthcare provider used her car to fetch them.

My mom has three siblings, one male and two females. My aunts are married and stay in Gauteng, and I don't blame them for not coming. My uncle stays around although he works in another province. Every month end he comes back home, unfortunately, he does not come to see me. (P6, M)

No one came, since I asked Matron S and he said I must wait because I just got admitted so even now I have not asked to talk to them nor ask them to visit me. I thought maybe you could try talking to them as you are one of the seniors in the hospital and your voice can be heard. (P11, M)

They are not giving me enough support and I just feel neglected because I am admitted but they are not coming to visit me or even call to check up on me. Since I was admitted here they never came, the only time they came was while I was still admitted at Hospital X after sister M insisted that they must come and visit me where she also used her car to bring all of them to the hospital. (P13, M)

#### Subtheme 2.2: Financial Challenges Perceived as a Source of Poor Support

Lack of finances is perceived as a source of poor support by participants. Arguments over finances result in family members not providing support to mental healthcare consumers. Some participants reported not attending follow-ups due to financial constraints. They linked poor finances with a lack of visits by family members. Participants raised their financial concerns with their families through the following quotes:

And there we were arguing about my issues and I asked for R20, they asked what I wanted to do with R20, and I said I wanted to go to Pietersburg to see my husband, that is when they became uncomfortable and decided to bring me back to the hospital. (P10, F)

Now, no one is coming to visit me, but my grandmother and my mother used to visit me the time I was admitted to the general hospital. Now they come once a month because of financial constraints because my aunt was retrenched at pick-n-pay, and my grandmother is a pensioner. (P2, M)

What I am saying is that they want me to give them my SASSA card and my identity document for them to use my money and when I refuse, they become angry hence they are not coming to visit me here at the hospital. They are not having money but what I hate is that they misuse my money whenever I give them my SASSA card. (P12, M)

Sometimes I would lack money to go to the hospital due to lack of finances and my mother was not supportive enough to take me to the hospital, she said she didn’t have money to assist me. (P13, M)

#### Subtheme 2.3: Lack of Psychological Support and Its Consequences

Family members can provide emotional and psychological support just through talking and listening. Participants reported not receiving psychological support from family members. Below are the quotes to attest to the statement above:

They say I eat too much, and they bully me with food. This traumatizes me. Sometimes I would not stay in the house due to their hatred and I would always be moving around in the streets since I don’t have a place to go. (P1, M)

No, they are not. If they can give me emotional support, help me recover from my condition, and accept me as their child maybe things can be better for me, even if I made mistakes in the past that does not mean that they disown me for good. I am hurt by the way they treat me. (P12, M).

My family thinks that I am crazy when I ask them to assist in solving my problems. I tried several times to handle my problems but things didn’t go well which is why I resorted to heavy drinking and smoking dagga. They think I am weak and irresponsible. I have accepted that because that is how they perceive me. (P13, M)

Participants indicated that sometimes they feel depressed due to how they are being treated. Below are the quotes that participants had to say during interviews that display symptoms of poor mental health:

I find myself so depressed that I feel stigmatized because they want to see everything I do, and they want to know when I'm out if I am not doing anything bad. Sometimes I get stressed when I think about how they treated me, they are holding grudges for me even though I did not hold grudges for what they did to me before. (P1, M).

No, she is not supportive, she just wants me to do good things for her when things are not going well she doesn’t support me she just wants me to be depressed. (P11, M)

## Discussion

The present study aimed to describe the MHCUs’ experiences of the family members’ knowledge of mental illness and support, thus identifying target areas for the development of guidelines to improve family members’ participation in the plan of care for consumers of mental health care. The findings revealed two themes and five subthemes. Knowledge is described as understanding, comprehension, or awareness acquired by experience, observation, or study (Collins Dictionary, 2024). Support means encouraging someone to succeed (Hajisadeghian et al., 2021).

Studies on mental health have been conducted but there is not enough information on the experiences of mental healthcare consumers regarding family members' knowledge of mental illness and support. In the study, participants alluded to the misconceptions about mental illness. Participants and their family members believed the cause of their mental illness was witchcraft. This could result in family members discouraging MHCUs from taking treatment and using alternate traditional medicine. The findings are consistent with Gavin and colleagues, who found that South African traditional health practitioners believed that persons suffering from mental illnesses are bewitched, or summoned to become traditional health practitioners themselves due to unhappy ancestors (Gavin et al., 2023).

Participants raised concerns about poor comprehension of mental disorders, the effect of medication, and the lack of support for MHCUs by family members. This is not surprising because globally, research has demonstrated poor knowledge of psychiatric conditions among consumers and family members (Bila & Carbonatto, 2022; Birkie & Anbesaw, 2021; Li & Reavley, 2021; Monnapula-Mazabane & Petersen, 2023). Family members are the most significant individuals in our lives. With poor comprehension of mental disorders in general and the importance of taking medication, family members fail to support MHCUs in treatment compliance (Raluthaga et al., 2023b). To be able to support their loved one, they must first become knowledgeable about the patient's condition. This is in line with Iswanti et al. (2023) who found that families with competent and accessible mental health workers to support and teach them how to care for their MHCUs and adaptive coping skills, make their caring experience simple and encouraging.

The study conducted by Borenstein (2020) discovered that knowledge and awareness are critical to understanding why our present experience with mental health differs from that of our family members. Therefore, with information about mental illness, both the consumers and family members will be able to understand the contributory factors to mental illness and the importance of taking treatment thereby taking treatment as prescribed rather than taking it anytime. As family members gain a better understanding of mental illness and how to support their loved ones, they may become the foundation of support for those suffering from mental illness. Addressing this knowledge deficit is essential to promote effective treatment adherence and the mental health of consumers.

In this study, participants expressed negative experiences of nonsupportive family members. In African culture, emotional, financial, and physical support is crucial. The absence of encouragement from loved ones might result in poor treatment compliance and service usage following release from the healthcare establishment (Raluthaga et al., 2023a). Participants believe that family members do not support them because of conflicts regarding their social grants. They reported that family members wanted to control their social grant and use it not for their benefit, and when consumers refused, were rejected. Financial hardship can have a direct impact on mental health and is linked with quality of life, stability, and social status and there can be a significant emotional toll.

A study by Iswanti et al. (2024) revealed that integrative empowerment had a significant positive impact on the family's capacity to care for and prevent relapses in individuals with Schizophrenia. The families showed an increase in identifying the symptoms of relapse, accepting their loved ones, and ensuring treatment adherence. Therefore, integrative empowerment can serve as an effective intervention for enhancing the quality of care and support that families provide to individuals with mental disorders.

The current study revealed that participants reported symptoms of poor psychological well-being like depression and stress due to the treatment they received from family members. Providing mental healthcare consumers with psychological care is important and can contribute to their speedy recovery and treatment adherence. According to Marklund and colleagues, receiving the assistance one needs as an individual would contribute to improved confidence (Marklund et al., 2020). Furthermore, loss of pleasure and emotional pain were found to be associated with unpleasant social encounters, which can be worsened by arguments over finances. Similarly, Wollburg et al. (2023) revealed providing financial support to healthcare users can improve mental health issues like depression and anxiety disorders which is reported by participants in the current study. Inadequate financial assistance for MHCUs might lead to low self-confidence and criminal behavior.

## Strengths and Limitations of the Study

This study used a purposive sample strategy, thus the findings may not be typical of the total population. When the interviews took place, the patients were in the hospital. The environment could have affected their responses. Despite these drawbacks, in-depth interviews allowed MHCUs to discuss their experiences with family members' knowledge of mental illness and support with minimal researcher interference. The incorporation of both interviews and observation produced rich results.

## Implication for Practice

Training family members on mental health illness is critical to the future of health care as there will be no misunderstanding between them and the MHCUs, and conflict over the social grant will be avoided thereby improving the consumers’ mental well-being. Conflicts over MHCUs' social grants should be avoided. Mental health policies should be developed/revised, and their implementation closely monitored. With little knowledge about the topic, a quantitative study can be done with discharged patients as a follow-up study to assess family members' knowledge of mental illness and support.

## Conclusion

Participants raised concerns about poor comprehension of mental disorders, the effect of medication, and the lack of support from family members. Therefore, integrative empowerment can serve as an effective intervention for enhancing the quality of care and support that families provide to individuals with mental disorders. The government should invest in assisting family members of mental healthcare consumers.
